# Development and Validation of a Non-invasive Model to Predict Liver Histological Lesions in Chronic Hepatitis B Patients With Persistently Normal Alanine Aminotransferase and Detectable Viremia

**DOI:** 10.3389/fmed.2022.944547

**Published:** 2022-07-13

**Authors:** Qiankun Hu, Qianqian Wang, Wei Xu, Chenlu Huang, Shuai Tao, Xun Qi, Yi Zhang, Xinyan Li, Xuhua Jiang, Jie Song, Qiang Li, Liang Chen, Yuxian Huang

**Affiliations:** ^1^Department of Liver Diseases, Shanghai Public Health Clinical Center, Fudan University, Shanghai, China; ^2^Department of Infectious Diseases, Shanghai Key Laboratory of Infectious Diseases and Biosafety Emergency Response, National Medical Center for Infectious Diseases, Huashan Hospital, Fudan University, Shanghai, China

**Keywords:** chronic hepatitis B, significant liver histological changes, persistently normal alanine aminotransferase, non-invasive model, antiviral therapy

## Abstract

**Background:**

A critical and controversial issue is whether antiviral therapy should be recommended in chronic hepatitis B virus (HBV) infection patients with persistently normal alanine aminotransferase (PNALT) and detectable HBV DNA. The study aimed to develop a non-invasive model for predicting significant liver histological changes (SLHC), which is the histological indication for antiviral therapy in chronic hepatitis B (CHB) patients with PNALT and detectable HBV DNA.

**Methods:**

398 chronic HBV infection patients with PNALT and detectable HBV DNA who underwent liver biopsy were divided into the estimation set (*n* = 256) and validation set (*n* = 142). A multivariate logistic regression model was developed to predict SLHC in the estimation set, and the diagnostic performance was further validated in the validation set.

**Results:**

132 patients (33.2%) with PNALT and detectable HBV DNA had SLHC. Aspartate aminotransferase (AST), cholinesterase (ChE), and liver stiffness measurement (LSM) were identified as the independent predictors of SLHC. The AUROC of the SLHC index, which combined AST, ChE, and LSM, was 0.824 and 0.816 in the estimation and validation set, respectively, for the prediction of SLHC. Applying the SLHC index ≤ 0.15, the presence of SLHC could be excluded with high negative predictive value in the estimation set (93.2%) and in the validation set (90.2%). Applying the SLHC index ≥ 0.55, the presence of SLHC could be considered with high positive predictive value in the estimation set (79.2%) and in the validation set (76.5%).

**Conclusion:**

The SLHC index provides a high accuracy in predicting liver histological indication for antiviral therapy in CHB patients with PNALT and detectable HBV DNA.

## Introduction

Chronic hepatitis B virus (HBV) infection remains a global public health issue, and approximately 240 million individuals are hepatitis B surface antigen (HBsAg) carriers worldwide (1). Chronic hepatitis B (CHB) patients with significant liver histological changes (SLHC), namely moderate to severe liver inflammation (METAVIR score ≥ A2) and/or significant liver fibrosis (METAVIR score ≥ F2), are considered to be at increased risk of disease progression to cirrhosis, hepatocellular carcinoma (HCC) and liver-related mortality (2). Effective antiviral therapy can improve prognosis by preventing inflammation and fibrosis progression, and consequently HCC development (2). Hence, it is critical for clinicians to identify CHB patients with SLHC and treat them as early as possible.

According to the guidelines on the management of CHB (2–4), the indications for initiating antiviral treatment for non-cirrhotic patients mainly depend on serum ALT, serum HBV DNA, and the severity of liver histological changes. The American Association for the Study of Liver Disease (AASLD) and the Asian Pacific Association for the Study of the Liver (APASL) guidelines recommend initiating antiviral treatment for patients with ALT > 2 times upper limit of normal (ULN) and elevated HBV DNA, whereas the European Association for the Study of the Liver (EASL) guideline is more liberal and recommends starting antiviral therapy in patients with ALT > ULN and elevated HBV DNA. For chronic HBV infection patients with normal ALT, it is generally believed that this proportion of patients are in either immune tolerant phase or inactive carrier phase. Considering the relatively low risk of disease progression and poor response to currently available treatments, the guidelines do not recommend antiviral therapy but close follow-up. Nevertheless, several studies showed that a considerable proportion of CHB patients with persistently normal ALT (PNALT) had SLHC, ranging from 25.4 to 38.2% (5–7). A meta-analysis concluded that approximately one fifth of CHB patients with ALT ≤ 40 IU/L had significant liver fibrosis (8). Clinical evidences also confirmed that chronic HBV infection patients with normal ALT were not exempted from disease progression to cirrhosis, or even HCC (9, 10). Therefore, it is a challenge to identify patients who are experiencing SLHC and in need of antiviral treatment in patients with PNALT and detectable HBV DNA.

Liver biopsy has traditionally been considered as the gold standard for assessment of liver histological lesion. However, the clinical application of liver biopsy is restricted by several limitations (11, 12). First, liver biopsy is a relatively expensive and invasive procedure. In addition, the low reproducibility and poor compliance make it difficult to evaluate the dynamic changes of liver histology. In the past few decades, numerous of non-invasive diagnostic methods, including serum models (13–15), transient elastography (TE) (16), and magnetic resonance elastography (MRE) (17), were developed as a surrogate to evaluate liver fibrosis and indeed helped a proportion of patients avoid liver biopsy. However, these non-invasive models had limited diagnostic value for liver inflammation.

There is a paucity of data on the diagnostic performance of non-invasive models for predicting histological indication for antiviral therapy, namely SLHC, in CHB patients with PNALT and detectable HBV DNA. In the present study, we aimed to develop a novel model index based on clinical frequently used parameters to predict the presence or absence of SLHC in CHB patients with PNALT and detectable HBV DNA.

## Materials and Methods

### Patients

**FIGURE 1:**
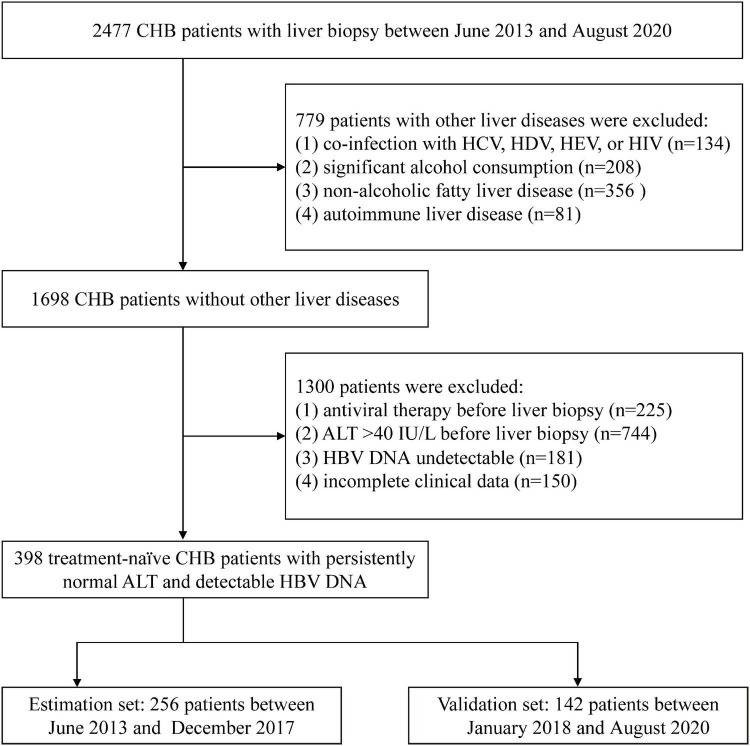
Flow diagram of the study population. CHB, chronic hepatitis B; HCV, hepatitis C virus; HDV, hepatitis D virus; HEV, hepatitis E virus; HIV, human immunodeficiency virus; ALT, alanine aminotransferase.

A total of 2,477 consecutive CHB patients who underwent liver biopsy in Shanghai Public Health Clinical Center, Shanghai, China, from June 2013 to August 2020, were retrospectively screened. CHB was defined as the persistent presence of serum HBsAg for more than 6 months. PNALT was defined as 3–4 consecutive detection of ALT ≤ ULN (40 IU/L) within 1 year. Exclusion criteria: (1) co-infection with HCV, HDV, HEV, or HIV (*n* = 134); (2) significant alcohol consumption (> 20 g/day for woman and > 30 g/day for men) (*n* = 208); (3) non-alcoholic fatty liver disease (*n* = 356); (4) autoimmune liver disease (*n* = 81); (5) antiviral therapy before liver biopsy (*n* = 225); (6) ALT > 40 IU/L before liver biopsy (*n* = 744); (7) HBV DNA undetectable (*n* = 181); (8) incomplete clinical data (*n* = 150). Eventually, 398 treatment-naïve chronic HBV infection patients with PNALT and detectable HBV DNA were enrolled. Patients who underwent liver biopsy between June 2013 and December 2017 comprised the estimation set, and patients who were biopsied between January 2018 and August 2020 comprised the validation set. The flow diagram of study population is shown in Figure 1.

### Laboratory Tests

Hematological markers including white bold cell, red blood cell, hemoglobin, and platelet were measured using Sysmex XT-2000i (Sysmex, Japan). Serum biochemical markers including ALT, aspartate aminotransferase (AST), *r*-glutamyl transpeptidase (GGT), alkaline phosphatase, cholinesterase (ChE), total bilirubin, albumin, globulin, and prealbumin were detected using biochemical analyzer 7600 Series (Hitachi, Japan). HBV serological markers including HBsAg and HBeAg were tested using Architect i2000 analyzer (Abbot Diagnostics, Chicago, IL, United States). Serum HBV DNA levels were quantified using real-time PCR (ABI 7500, Applied Biosystems, Foster City, CA, United States) with a lower detection limit of 100 IU/mL.

### Liver Histological Assessment

Percutaneous liver biopsy was performed using 16-gauge needles. The liver samples were fixed with 10% formalin and then embedded in paraffin. Hematoxylin and eosin, reticular fiber and Masson’s trichrome staining were processed to evaluate the liver histology. The liver specimens were considered sufficient for histological analysis with a minimum length of 15 mm and at least 6 portal tracts. All biopsy samples were examined independently by two experienced pathologists who were blinded to patients’ characteristics. When discrepancies occurred, the specimens were re-examined by a third senior pathologist, and the three pathologists gave a final score after joint discussion. The stage of liver necroinflammation and fibrosis were analyzed according to the METAVIR scoring system (18). Liver inflammation included four grades: A0, none inflammatory activity; A1, mild inflammatory activity; A2, moderate inflammatory activity; and A3, severe inflammatory activity. Liver fibrosis included five stages: F0, no fibrosis; F1, portal fibrosis without septa; F2, portal fibrosis with rare septa; F3, numerous septa without cirrhosis; and F4, cirrhosis. A2-3 and F2-4 were considered moderate to severe inflammation and fibrosis, respectively. According to current CHB guidelines (2–4), patients who had moderate to severe inflammation (≥ A2) and/or fibrosis (≥ F2) fulfilled the histological indication for antiviral therapy.

### Non-invasive Liver Histological Tests

Liver stiffness measurement (LSM) was evaluated by transient elastography (FibroScan, Echosens, Pairs, France) prior to liver biopsy. The value of LSM was considered reliable fulfilling the following criteria: 10 validated measurements, LSM success rate above 60% and an interquartile range of less than 30% of the median elasticity (16). The calculation formulae of commonly used non-invasive models including the AST to PLT ratio (APRI) (13), the fibrosis index based on four factors (FIB-4) (14), and the GGT to PLT ratio (GPR) (15) are shown as following:


$$\text{APRI}=\frac{\text{AST}\;(\text{IU}/\text{L})/\mathrm{ULN}\;\mathrm{of}\;\mathrm{AST}}{\text{PLT}\;(10^{9}/\text{L})}\times 100$$

$$\mathrm{FIB}-4=\frac{\text{Age}\;(\text{years})\times \mathrm{AST}\;\left(\text{IU}/\text{L}\right)}{\text{PLT}\;(10^{9}/\text{L})\times \sqrt{\text{ALT}\;(\text{IU}/\text{L})}}$$

$$\text{GPR}=\frac{\text{GGT}\;(\text{IU}/\text{L})/\mathrm{ULN}\;\mathrm{of}\;\mathrm{GGT}}{\text{PLT}\;(10^{9}/\text{L})}\times 100$$

In the formulae, the ULN of AST and GGT is 40 IU/L and 60 IU/L, respectively.

### Statistical Analysis

Quantitative variables were presented as mean ± standard deviation or median [interquartile range (IQR)], and categorical variables were presented as number (percentage). Difference between groups was analyzed using the *t*-test or Mann-Whitney *U* test for continuous parameters, and the Chi-square test or Fisher’s exact test for categorical parameters. Logistic regression analysis was performed to identify variables associated with SLHC. The diagnostic performances of non-invasive models were evaluated using the area under the receiver operating characteristic curves (AUROC). The diagnostic accuracy was evaluated using sensitivity, specificity, positive predictive value (PPV), and negative predictive value (NPV). All tests were two-sided, and *p*-value < 0.05 was considered statistically significant. Statistical analysis was performed using SPSS software version 25.0 (Chicago, IL, United States) and MedCalc software version 15.2.2 (MedCalc, Mariakerke, Belgium).

## Results

### Study Population

**FIGURE 2:**
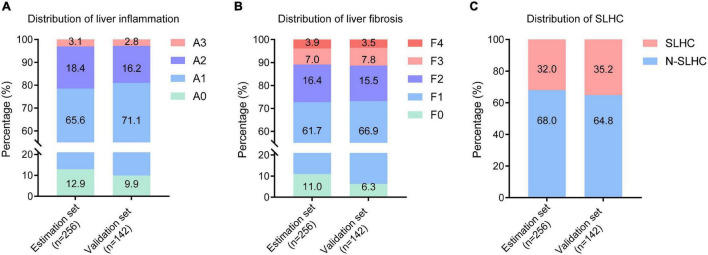
Distribution of liver inflammation, fibrosis and SLHC among enrolled patients. **(A)** Distribution of liver inflammation in the estimation and validation set; **(B)** Distribution of liver fibrosis in the estimation and validation set; **(C)** Distribution of liver histological change in the estimation and validation set. SLHC, significant liver histological changes; N-SLHC, non-significant liver histological changes.

The demographic, laboratory, and liver histological characteristics of the study population were presented in Table 1 and Figure 2. Among the 398 enrolled patients, the median age was 39 (IQR, 32–46) years; 234 patients (58.8%) were male, and 156 patients (39.2%) were HBeAg positive. The median HBsAg, HBV DNA, and ALT was 3.51 (IQR, 3.07–3.98) log_10_IU/mL, 3.85 (IQR, 2.86–6.63) log_10_IU/mL, and 27 (IQR, 19–34) IU/L, respectively. According to the METAVIR scoring system, 55 of 256 patients (21.5%) and 27 of 142 patients (19.0%) had moderate to severe inflammation in the estimation set and validation set, respectively; 70 of 256 patients (27.3%) and 38 of 142 patients (26.8%) had significant liver fibrosis in the estimation set and validation set, respectively. A total of 82 patients (32.0%) in the estimation set and 50 patients (35.2%) in validation set were identified with SLHC (Figure 2). There was no significant difference between the estimation and validation set in any of studied parameters.

**TABLE 1:** Characteristics of the study population.

Variables	Total (*n* = 398)	Estimation set (*n* = 256)	Validation set (*n* = 142)	*P*-value^‡^
Age (years)	39 (32–46)	39 (32–47)	38 (33–45)	0.314
Male, *n* (%)	234 (58.8%)	147 (57.4%)	87 (61.3%)	0.455
HBeAg positive, *n* (%)	156 (39.2%)	95 (37.1%)	61 (43.0%)	0.252
HBsAg (log_10_ IU/mL)	3.51 (3.07–3.98)	3.51 (3.06–3.98)	3.52 (3.07–3.98)	0.643
HBV DNA (log_10_ IU/mL)	3.85 (2.86–6.63)	3.94 (2.79–6.44)	3.80 (3.17–6.84)	0.338
White blood cell (10^9^/L)	5.5 (4.6–6.4)	5.6 (4.7–6.5)	5.3 (4.5–6.3)	0.145
Red blood cell (10^12^/L)	4.7 (4.3–5.1)	4.7 (4.3–5.2)	4.7 (4.3–5.1)	0.747
Hemoglobin (g/L)	145 (132–157)	144 (132–157)	147 (130–156)	0.935
Platelet (10^9^/L)	186 (156–220)	188 (156–221)	182 (155–215)	0.463
ALT (IU/L)	27 (19–34)	26 (19–33)	30 (20–34)	0.159
AST (IU/L)	24 (20–29)	24 (19–29)	25 (20–30)	0.116
ALP (IU/L)	70 (60–85)	69 (60–85)	71 (59–86)	0.889
GGT (IU/L)	22 (16–32)	23 (16–33)	21 (16–30)	0.261
ChE (kU/L)	8.8 (7.6–10.0)	8.9 (7.6–10.2)	8.6 (7.5–9.9)	0.330
Total bilirubin (μmol/L)	12.9 (9.4–16.8)	13.0 (10.2–16.7)	12.5 (9.2–16.8)	0.245
Albumin (g/L)	43.8 ± 3.6	43.9 ± 3.5	43.8 ± 3.9	0.694
Globulin (g/L)	30.1 ± 4.0	30.4 ± 4.0	29.7 ± 3.9	0.085
Prealbumin (mg/L)	233 (194–278)	234 (189–284)	231 (200–265)	0.607
AFP (ng/mL)	2.6 (1.9–3.8)	2.6 (1.9–3.8)	2.8 (2.1–4.2)	0.335
LSM (kPa)	6.4 (5.2–8.1)	6.3 (5.2–8.1)	6.5 (5.3–8.2)	0.447
**Liver inflammation grade, *n* (%)**				
A0	47 (11.8%)	33 (12.9%)	14 (9.9%)	0.708
A1	269 (67.6%)	168 (65.6%)	101 (71.1%)	
A2	70 (17.6%)	47 (18.4%)	23 (16.2%)	
A3	12 (3.0%)	8 (3.1%)	4 (2.8%)	
**Liver fibrosis stage, *n* (%)**				
F0	37 (9.3%)	28 (11.0%)	9 (6.3%)	0.625
F1	253 (63.5%)	158 (61.7%)	95 (66.9%)	
F2	64 (16.1%)	42 (16.4%)	22 (15.5%)	
F3	29 (7.3%)	18 (7.0%)	11 (7.8%)	
F4	15 (3.8%)	10 (3.9%)	5 (3.5%)	
Moderate to severe inflammation (≥ A2), *n* (%)	82 (20.6%)	55 (21.5%)	27 (19.0%)	0.559
Significant fibrosis (≥ F2), *n* (%)	108 (27.1%)	70 (27.3%)	38 (26.8%)	0.900
SLHC (≥ A2 and/or ≥ F2), *n* (%)	132 (33.2%)	82 (32.0%)	50 (35.2%)	0.519

*ALT, alanine aminotransferase; AST, aspartate aminotransferase; ALP, alkaline phosphatase; GGT, γ-glutamyl transpeptidase; ChE, cholinesterase; AFP, alpha fetoprotein; LSM, liver stiffness measurement; SLHC, significant liver histological changes. ^‡^P-values were calculated between the estimation set and validation set.*

### Construction of a Novel Non-invasive Model for the Prediction of Significant Liver Histological Changes

In the estimation set, patients were divided into two groups based on the results of liver biopsy: N-SLHC group and SLHC group. Logistic regression analysis was performed to investigate independent parameters associated with SLHC. Univariate analysis revealed that platelet, AST, ALP, GGT, ChE, albumin, prealbumin, AFP, and LSM were significantly different in the two groups (all *p* < 0.05) (Table 2). Subsequent multivariate regression analysis identified AST (*p* = 0.007), ChE (*p* = 0.001), and LSM (*p* < 0.001) as independent predictors of SLHC (Table 3). Consequently, a novel model index consisting of AST, ChE, and LSM, named as the SLHC index, was constructed according to binary logistic regression analysis with forward stepwise selection:


Y=0.056*AST(IU/L)−0.362*ChE (kU/L)+0.462*LSM (kPa)−2.283

The SLHC index=exp(Y)/[1+exp(Y)]

**TABLE 2:** Variables associated with the presence of SLHC by univariate analysis in the estimation set.

Variables	Non-SLHC (*n* = 174)	SLHC (*n* = 82)	*P*-value
Age (years)	38 (32–46)	42 (32–48)	0.311
Male, *n* (%)	102 (58.6%)	45 (54.9%)	0.572
HBeAg, Positive, *n* (%)	62 (35.6%)	33 (40.2%)	0.476
HBsAg (log_10_ IU/mL)	3.55 (3.05–4.14)	3.42 (3.09–3.79)	0.169
HBV DNA (log_10_ IU/mL)	3.79 (2.76–6.58)	4.18 (2.81–6.06)	0.767
White blood cell (10^9^/L)	5.6 (4.7–6.6)	5.5 (4.8–6.2)	0.546
Red blood cell (10^12^/L)	4.8 (4.4–5.2)	4.7 (4.3–4.9)	0.118
Hemoglobin (g/L)	146 (135–158)	142 (129–156)	0.167
Platelet (10^9^/L)	192 (161–230)	177 (148–205)	<0.001
ALT (IU/L)	26 (19–33)	28 (19–34)	0.334
AST (IU/L)	22 (19–26)	28 (23–33)	<0.001
ALP (IU/L)	68 (60–83)	76 (63–90)	0.004
GGT (IU/L)	22 (15–30)	26 (19–40)	0.006
ChE (kU/L)	9.1 (8.0–10.7)	7.8 (6.8–9.3)	<0.001
Total bilirubin (μmol/L)	12.9 (10.3–16.4)	13.9 (9.4–17.0)	0.502
Albumin (g/L)	44.2 ± 3.3	43.1 ± 3.9	0.023
Globulin (g/L)	30.3 ± 3.9	30.6 ± 4.2	0.571
Prealbumin (mg/L)	247 (202–297)	208 (171–257)	<0.001
AFP (ng/mL)	2.5 (1.9–3.6)	3.1 (2.0–5.7)	0.005
LSM (kPa)	5.8 (4.8–7.1)	8.1 (6.3–10.1)	<0.001

*SLHC, significant liver histological changes; ALT, alanine aminotransferase; AST, aspartate aminotransferase; ALP, alkaline phosphatase; GGT, γ-glutamyl transpeptidase; ChE, cholinesterase; AFP, alpha fetoprotein; LSM, liver stiffness measurement.*

**TABLE 3:** Predictors of SLHC by multivariate analysis with forward stepwise selection in the estimation set.

Parameter	Coefficient	Exp (B)	95% CI	*P*-value
AST (IU/L)	0.056	1.058	1.015–1.102	0.007
ChE (kU/L)	–0.362	0.696	0.580–0.836	<0.001
LSM (kPa)	0.462	1.588	1.344–1.876	<0.001
Constant	–2.283	0.102	–	0.041

*SLHC, significant liver histological changes; AST, aspartate aminotransferase; ChE, cholinesterase; LSM, liver stiffness measurement; CI, confidence interval.*

### AUROCs Comparison of Non-invasive Tests for the Prediction of Significant Liver Histological Changes

**FIGURE 3:**
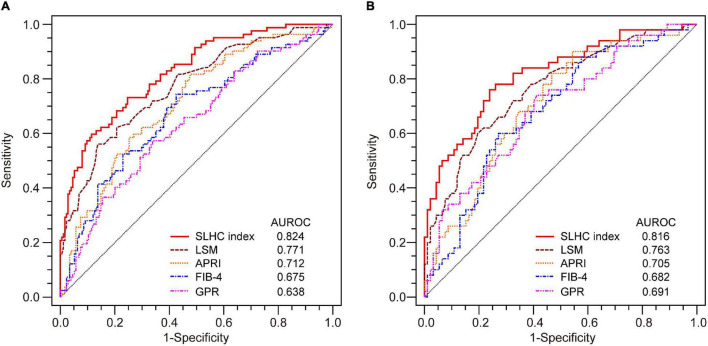
AUROCs comparison of non-invasive tests for the prediction of SLHC. **(A)** In the estimation set, the AUROC of the SLHC index was significantly higher than that of LSM (0.824 vs. 0.771, *p* = 0.021), APRI (0.824 vs. 0.712, *p* < 0.001), FIB-4 (0.824 vs. 0.675, *p* < 0.001), and GPR (0.824 vs. 0.638, *p* < 0.001). **(B)** In the validation set, the AUROC of the SLHC index was also significantly higher than that of LSM (0.816 vs. 0.763, *p* = 0.046), APRI (0.816 vs. 0.705, *p* = 0.014), FIB-4 (0.816 vs. 0.682, *p* = 0.003), and GPR (0.816 vs. 0.691, *p* = 0.005). SLHC, significant liver histological changes; LSM, liver stiffness measurement; APRI, aspartate aminotransferase to platelet ratio; FIB-4, fibrosis index based on four factors; GPR, *r*-glutamyl transpeptidase to platelet ratio; AUROC, the area under the receiver operating characteristic curve; CI, confidence interval.

AUROCs comparison of non-invasive tests for the prediction of SLHC is shown in Table 4 and Figure 3. The SLHC index yielded an AUROC of 0.824 (95% CI 0.772–0.869) in the estimation set and 0.816 (95% CI 0.742–0.876) in the validation set for predicting SLHC. In the estimation set, the AUROC of the SLHC index was significantly higher than that of LSM (0.824 vs. 0.771, *p* = 0.021), APRI (0.824 vs. 0.712, *p* < 0.001), FIB-4 (0.824 vs. 0.675, *p* < 0.001), and GPR (0.824 vs. 0.638, *p* < 0.001). In the validation set, the AUROC of the SLHC index was also significantly higher than that of LSM (0.816 vs. 0.763, *p* = 0.046), APRI (0.816 vs. 0.705, *p* = 0.014), FIB-4 (0.816 vs. 0.682, *p* = 0.003), and GPR (0.816 vs. 0.691, *p* = 0.005).

**TABLE 4:** AUROCs comparison of non-invasive tests for the prediction of SLHC.

	Estimation set		Validation set	
	AUROC	95% CI	AUROC	95% CI
SLHC index	0.824	0.772–0.869	0.816	0.742–0.876
LSM	0.771	0.714–0.821	0.763	0.685–0.831
APRI	0.712	0.652–0.767	0.705	0.623–0.779
FIB-4	0.675	0.614–0.732	0.682	0.598–0.757
GPR	0.638	0.575–0.696	0.691	0.608–0.765
**Comparison of AUROCs**				
SLHC index vs. LSM	*p* = 0.021		*p* = 0.046	
SLHC index vs. APRI	*p* < 0.001		*p* = 0.014	
SLHC index vs. FIB-4	*p* < 0.001		*p* = 0.003	
SLHC index vs. GPR	*p* < 0.001		*p* = 0.005	

*SLHC, significant liver histological changes; LSM, liver stiffness measurement; APRI, aspartate aminotransferase to platelet ratio; FIB-4, fibrosis index based on four factors; GPR, r-glutamyl transpeptidase to platelet ratio; AUROC, the area under the receiver operating characteristic curve; CI, confidence interval.*

The SLHC index was further evaluated separately in HBeAg positive patients with normal ALT and high HBV DNA levels (phase 1 of the EASL guidelines) and in HBeAg negative patients with normal ALT and low viral loads (phase 3 of the EASL guidelines). The results showed that the performance of the SLHC index in HBeAg positive patients was slightly better than that in HBeAg negative patients both in the estimation set (AUROC, 0.856 vs. 0.810) and in the validation set (AUROC, 0.844 vs. 0.806) (Supplementary Table 1).

### Cut-Off Values and Diagnostic Accuracy of the Significant Liver Histological Changes Index for the Prediction of Significant Liver Histological Changes

The cut-off value and diagnostic accuracy of the SLHC index for the prediction of SLHC is shown in Table 5. Based on the ROC analysis, a low cut-off value (≤ 0.15) was chosen by obtaining a sensitivity of at least 90%, and a high cut-off value (≥ 0.55) was chosen by obtaining a specificity of at least 90%. Applying the SLHC index ≤ 0.15, the presence of SLHC could be excluded with a high NPV of 93.2% (82/88) in the estimation set and 90.2% (37/41) in the validation set. Applying the SLHC index ≥ 0.55, the presence of SLHC could be identified with a high PPV of 79.2% (38/48) in the estimation set and 76.5% (26/34) in the validation set.

**TABLE 5:** Cut-off value and diagnostic accuracy of the SLHC index for the prediction of SLHC.

			Liver biopsy		Se%	Sp%	PPV%	NPV%
			Non-SLHC	SLHC				
Estimation set	Low cut-off				92.7	47.1	45.2	93.2
	≤ 0.15	88	82	6				
	> 0.15	168	92	76				
	High cut-off				46.3	94.3	79.2	78.8
	< 0.55	208	164	44				
	≥ 0.55	48	10	38				
Validation set	Low cut-off				92.0	40.2	45.5	90.2
	≤ 0.15	41	37	4				
	> 0.15	101	55	46				
	High cut-off				52.0	91.3	76.5	77.8
	< 0.55	108	84	24				
	≥ 0.55	34	8	26				

*SLHC, significant liver histological changes; Se, sensitivity; Sp, specificity; PPV, positive predictive value; NPV, negative predictive value.*

## Discussion

This study revealed that appropriately one third of patients with PNALT and detectable HBV DNA had SLHC, which is considered as the histological indication for anti-HBV therapy. Multivariate analysis identified AST, ChE, and LSM as the independent predictors of SLHC. A novel model index consisting of AST, ChE, and LSM, named as the SLHC index, was developed to predict SLHC, which yielded an AUROC of 0.824 and 0.816 in the estimation set and validation set, respectively. The presence of SLHC could be correctly excluded in patients with the SLHC index ≤ 0.15 with a high NPV (90.2–93.2%), and the presence of SLHC could be correctly considered in patients with the SLHC index ≥ 0.55 with a high PPV (76.5–79.2%). Thus, the SLHC index could be used as a risk stratification tool of SLHC in CHB patients with PNALT and detectable HBV DNA.

Serum ALT is a sensitive biochemical marker reflecting liver injury. According to current guidelines, the elevated ALT is recommended as a serological indicator for antiviral therapy (2–4). In the traditional ideas, CHB patients with PNALT are considered to be at relatively low risk of adverse clinical outcomes. However, a growing number of evidences confirmed that patients with PNALT are not completely free from liver histological damage (5–7). Consistent with previous studies, this study found that 82 of 398 patients (20.6%) had moderate to severe liver inflammation, 108 of 398 patients (27.1%) had significant liver fibrosis, and 132 of 398 patients (33.2%) had SLHC. The potential causes of SLHC in patients with PNALT are shown as following: (1) the natural history of chronic HBV infection is characterized by intermittent fluctuations in ALT and HBV DNA (19), and patients with SLHC may experience spontaneous normalization of ALT; (2) the commonly used ULN of ALT (40 IU/L) might be high for patients with CHB. Prati and colleagues (20) evaluated the ALT levels in a large number of first-time blood-donor candidates and proposed to adjust the ULN of ALT to 30 IU/L for men and 19 IU/L for women. The EASL guidelines in 2017 (2) and the updated AASLD guidelines in 2018 (3) also recommended to redefine the normal range of serum ALT.

In this study, we indicated that AST is one of the independent predictors of SLHC, which is consistent with previous studies. Wu et al. (21) reported that AST was one of the independent variables for antiviral therapy decision-making with an AUROC of 0.718 in CHB patients with ALT < 2 ULN. Cheong and colleagues (22) found a positive correlation (*r* = 0.445, *p* < 0.001) between serum AST levels and liver inflammatory activities in CHB or CHC patients with ALT ≤ 60 IU/L, and AST yielded the highest performance (AUROC = 0.784) for predicting significant inflammation compared with other clinical variables. Wang et al. (23) reported that AST was one of independent predictors of significant fibrosis with an AUROC of 0.73 in CHB patients with ALT < 2 ULN. The above-mentioned studies indicated that AST could provide additional information for chronic liver damage when serum ALT level was normal or mildly elevated.

In this study, ChE was identified as one of the independent predictors of SLHC. ChE is an enzyme mainly synthesized in hepatocytes and released into circulation, and a decreased serum ChE level usually reflects the impairment of liver synthetic function (24). The detection of serum ChE might be a cost-effective approach to distinguish between overt liver injury and other clinical factors of abnormal liver function tests (25). A study of 2343 CHB patients revealed that serum ChE was closely correlated with liver fibrosis, and the GCPR model including ChE presented better diagnostic performance than GPR in differentiating significant fibrosis and cirrhosis (26). Wu et al. (27) also reported that serum ChE was an independent indicator of advanced fibrosis in CHB patients. Of note, serum ChE activity can be affected by several factors, such as obesity, diabetes, uremia, hyperthyroidism, and lipid metabolism disorder (24). Therefore, the above physiological and pathological conditions should be taken into account when evaluating ChE value as a variable associated with SLHC.

The good diagnostic performance of LSM for the prediction of liver fibrosis and cirrhosis in CHB patients has been validated in several studies (28–30). Moreover, the strategies of combining LSM with serum markers could further improve the diagnostic accuracy for significant fibrosis in CHC or CHB patients (31, 32). Similarly, our study also showed that the SLHC index, which combined AST, ChE, and LSM, presented a higher diagnostic accuracy than LSM for the prediction of SLHC. Notably, considering that the diagnostic accuracy of LSM might be influenced by elevated ALT and steatosis, the LSM cut-off values stratified by serum ALT levels have been proposed for the prediction of liver fibrosis in CHB patients (33). In this study, all patients had PNALT, which minimized the influence of inflammatory activity on the diagnostic performance of LSM. Actually, a study revealed that the diagnostic performance of LSM was not enhanced by ALT-stratified cut-off values in CHB patients with normal or mildly elevated ALT (34).

The SLHC index has several attractive features of non-invasiveness and relatively low cost. More importantly, compared with other non-invasive models, the SLHC index presented significantly better diagnostic performance in distinguishing patients with or without SLHC. Of note, in this study, the AUROCs of APRI, FIB-4, and GPR were lower than previously published data. A meta-analysis reported that the AUROCs of APRI and FIB-4 were 0.741 and 0.784, respectively, for the diagnosis of significant liver fibrosis (35). A large-sample study revealed that the AUROCs of GPR were 0.67 and 0.71, respectively, for the prediction of significant liver fibrosis and cirrhosis in chronic HBV infection patients (36). The discrepancy might be explained by the difference in participants. Patients in this study had PNALT, whereas previous studies included a considerable proportion of patients with elevated ALT. Besides, in this study, the APRI, FIB-4, and GPR were evaluated to predict SLHC, rather than liver fibrosis. Given that APRI, FIB-4, and GPR had limited diagnostic accuracy in liver inflammation, it might result in lower AUROCs for predicting SLHC compared with predicting fibrosis.

There are several limitations in our study. Firstly, this is a retrospective study, which might cause selective bias. Multicenter prospective cohort studies are necessary to further validate the clinical application of the SLHC index. Secondly, in this study, 46.9% patients (120/256) in the estimation set and 47.2% patients (67/142) in the validation set had the SLHC index between 0.15 and 0.55, which indicated that almost one half of patients could not directly benefit from the SLHC index. Although the SLHC index cannot replace liver biopsy, it can select the candidates for liver biopsy, avoid excessive liver biopsy, and narrow down the group which really needs liver biopsy. Thirdly, we could not compare the diagnostic performance of the SLHC index with that of the patented non-invasive models such as Fibrotest, Hepascore, and Fibrometer.

In conclusion, this study revealed that appropriately one third of patients with PNALT and detectable HBV DNA fulfilled histological criteria for anti-HBV therapy. A novel non-invasive model, named as the SLHC index, provided a high accuracy for the prediction of SLHC, and could be used as a risk stratification tool of SLHC in CHB patients with PNALT and detectable HBV DNA.

## Data Availability Statement

The original contributions presented in this study are included in the article/Supplementary Material, further inquiries can be directed to the corresponding authors.

## Ethics Statement

The study was approved by the Clinical Research Ethics Committee of the Shanghai Public Health Clinical Center. All patients provided informed consent prior to the study, and the procedures followed were in accordance with the ethical standards of the Helsinki Declaration (1964, amended most recently in 2008) of the World Medical Association.

## Author Contributions

QH, QW, WX, and QL collected the data, analyzed the data, and wrote the manuscript. CH, ST, XQ, YZ, XL, XJ, and JS collected the data. YH, LC, and QL designed the study and revised the manuscript. All authors read and approved the final manuscript.

## Conflict of Interest

The authors declare that the research was conducted in the absence of any commercial or financial relationships that could be construed as a potential conflict of interest.

## Publisher’s Note

All claims expressed in this article are solely those of the authors and do not necessarily represent those of their affiliated organizations, or those of the publisher, the editors and the reviewers. Any product that may be evaluated in this article, or claim that may be made by its manufacturer, is not guaranteed or endorsed by the publisher.
