# Supplementary material for: Development and Validation of a Non-invasive Model to Predict Liver Histological Lesions in Chronic Hepatitis B Patients With Persistently Normal Alanine Aminotransferase and Detectable Viremia
Source: Front Med (Lausanne). 2022 Jul 13;9:944547. doi: 10.3389/fmed.2022.944547 (PMC9326251; doi:10.3389/fmed.2022.944547)
Supplement: Supplementary file 1 [file Table_1.DOCX]

**Supplementary Materials:**

**Table S1.** AUROCs of noninvasive tests for the prediction of SLHC by HBeAg status

|  |  | HBeAg positive patients | |  | HBeAg negative patients | |
| --- | --- | --- | --- | --- | --- | --- |
|  |  | AUROC | 95% CI |  | AUROC | 95% CI |
| Estimation set | SLHC index | 0.856 | 0.768-0.916 |  | 0.810 | 0.738-0.865 |
|  | LSM | 0.785 | 0.687-0.862 |  | 0.764 | 0.701-0.826 |
|  | APRI | 0.717 | 0.616-0.805 |  | 0.703 | 0.632-0.778 |
|  | FIB-4 | 0.679 | 0.575-0.771 |  | 0.672 | 0.623-0.727 |
|  | GPR | 0.687 | 0.584-0.779 |  | 0.626 | 0.568-0.702 |
| Validation set | SLHC index | 0.844 | 0.728-0.924 |  | 0.806 | 0.703-0.885 |
|  | LSM | 0.803 | 0.702-0.892 |  | 0.741 | 0.632-0.825 |
|  | APRI | 0.697 | 0.566-0.808 |  | 0.717 | 0.606-0.812 |
|  | FIB-4 | 0.736 | 0.607-0.841 |  | 0.632 | 0.518-0.736 |
|  | GPR | 0.712 | 0.581-0.820 |  | 0.650 | 0.533-0.752 |

SLHC, significant liver histological changes; LSM, liver stiffness measurement; APRI, aspartate aminotransferase to platelet ratio; FIB-4, fibrosis index based on four factors; GPR, *r*-glutamyl transpeptidase to platelet ratio; AUROC, the area under the receiver operating characteristic curve; CI, confidence interval.
